# Incidence and Risk Factors of Workplace Violence on Nursing Staffs Caring for Chronic Psychiatric Patients in Taiwan

**DOI:** 10.3390/ijerph6112812

**Published:** 2009-11-12

**Authors:** Wen-Ching Chen, Yu-Hua Sun, Tsuo-Hung Lan, Hsien-Jane Chiu

**Affiliations:** Yuli Hospital, Department of Health, Executive Yuan, Taiwan / No.448, Jhonghua Rd., Yuli Township, Hualien County 981, Taiwan; E-Mails: hilda@ms28.url.com.tw (Y.H.S.); tosafish@ms73.hinet.net (T.H.L.); dire@mail.ttyl.doh.gov.tw (H.J.C.)

**Keywords:** workplace violence, incidence, nursing staff, risk factor, psychiatry

## Abstract

This one-year follow-up study determined the incidence and risk factors of workplace violence against nursing staff in a psychiatric hospital. The cohort members had a website to report events whenever they came across violence. A total of 971 events were reported. The incidence rates of physical violence, verbal abuse, bullying/mobbing, sexual harassment, and racial harassment were 1.7, 3.7, 0.2, 0.3, and 0 per staff-year, respectively. Young age, female sex, lower education, shorter duration of employment, and high level of anxiety of staff seemed to be the determinants of violence. Pre-placement education should focus on these staff to reduce workplace violence.

## 1. Introduction

Violence is a global problem. In 1996, the 49^th^ United Nations World Health Committee announced that prevention of violence is a leading priority for public health researchers and practitioners [1]. Based on World Health Organization statistics, more than 16 million people worldwide die each year from violence. Among 15–44 year olds, violence is the leading cause of death, making up 14% of male and 7% of female deaths in this group [1]. Before 1990, workplace violence was largely ignored and underestimated. Changing society and increasing public awareness and standards of public concern have shifted attention from traditional workplace dangers by physical, chemical, radiological, biological, and ergonomic causes, to focus on psycho-sociological harm, such as workplace violence, sexual harassment, sudden death from overexertion, and work pressure [2,3]. Since workplace harassment, including physical violence (PV), verbal abuse (VA), bullying/mobbing (BM), sexual harassment (SH), and racial harassment (RH), has increased in recent years, in 2002, the United States National Institute for Occupational Safety and Health (NIOSH) vigorously promoted the hope that people around the world would take note of increasing workplace violence [4]. The US Department of Labor, Occupational Safety and Health Administration (OSHA) has also made an effort to establish guidelines for the prevention of workplace violence [5].

Regarding to the national case studies conducted in Australia, Brazil, Bulgaria, and so on, in general hospitals [6] found that the annual prevalence rates of physical PV ranged from 3% to 17%, VA 27.4% to 67%, BM 10.5% to 23%, SH 0.7% to 8%, and RH 0.8% to 2.7%. Using different questionnaires, research conducted in England [7], Hong Kong [8] and China [9] found that PV varied from 5.3% to 21% and VA from 43% to 73%.

NIOSH notes that the public place where most such violence can be observed is in hospitals, especially psychiatry wards, emergency rooms, waiting rooms, and geriatric wards [4]. In psychiatry hospitals, the primary care staffs, nurses and nurse aides, are particularly easy targets for psychiatric patients [10,11]. In Taiwan, according to a questionnaire survey conducted on 2003, about 61% of nurses and nurse aides reported being attacked by patients within previous one year in this hospital, but the incidence was still unknown.

The risk factors of workplace violence were largely explored in the environmental and patient factors. Viitasara declared that violence was influenced by underlying structural and situational risk factors [12]. Regarding patient profiles, Lowenstein, McNiel, and Nijman stated that patients manifesting higher levels of thinking disturbances, hostile-suspiciousness and agitation-excitement presented higher risk of violence [13–15]. Tiihonen and Wallace found that alcohol-induced psychosis, schizophrenia with alcohol abuse, or co-morbided with personality disorder and substance abuse increased offending behaviors [16,17]. Nevertheless, the risk factors for primary care staff were relatively less explored. Anderson found that staff with abuse history was more vulnerable to physical violence and sexual harassment [18]. Binder said that the role relationship with patients, not the gender was a predictor of violence [10]. In Taiwan, Chou found that young age and shorter duration of employment were the risk factors for violence towards nurses in acute psychiatric wards through a questionnaire survey [19].

The Internet, which is speeding up the progress of every sphere of human life, is now commonly used in Taiwan. A study revealed that the convenience and privacy of the Internet increased the motivation of some people to seek help [20]. The reliability of information provided by clients was high [21]. In addition, a study showed that the questionnaire survey from a website was as reliable as from paper form [22]. Therefore, we used the web-form report system to conduct a one-year follow-up study to determine the incidence rate and risk factors of workplace violence encountered by nursing staff caring for chronic psychiatric patients in Taiwan.

## 2. Methods

### 2.1. The Hospital and Staff

The hospital in our study was located in a rural area in eastern Taiwan which had emerged from an asylum established in 1965. The asylum was a governmental institution aimed to take long-term care of homeless or chronic psychiatric patients. In the early days there were only a few medical doctors and nurses to take care of the patients’ psychiatric and medical problems. The administration of the asylum was under a veteran’s hospital which was operated more as a military facility than a hospital. In the 1990s, partly due to the pressures from human rights groups, the asylum became independent from the veteran’s hospital and was reestablished as a modern psychiatric hospital. Currently, it has more than 2,700 psychiatric in-patients, about 85% of them with diagnoses of schizophrenia. The average admission of patients over 65 years of age in the hospital was about 16 years [23]. According to their mental status they were arranged to live in different settings, including two acute wards, seven chronic wards and four rehabilitation centers. There were about 200 psychiatric nurses and nurse aides employed during data collection for this study. The main task of the nursing aides in this hospital is helping nurses with medical care, instructing and supervising patients in daily life activities, and keeping the environment clean, and they are in direct contact with patients, just all the same as nurses are [24].

### 2.2. Study Procedure

This study followed the Helsinki Declarations. Before it began we had sent out a protocol describing the purposes, methods of data collection, uses of data, and guarantees for the privacy of participants to the institutional bioethics committee charged with permitting this research and we got their approval. Then we set up a reporting system on the website where the cohort members could report violent events after they had obtained their account and password. After we were certain that the reporting system was working well, we recruited a total of 174 nurse and nursing staff volunteers into this study; the participant rate was about 87%. The reasons of non-participation included that they were too busy; they did not know the study; they could not use the computer. All of participants signed an informed consent and practiced the procedure until they could access the website smoothly and complete the report correctly. On the website, we first put a basic questionnaire which included demographics, working history, and the anxiety level using a question as “how you fear the threat of workplace violence caused by patients?”. This question was taken from a questionnaire developed by ILO/ICN/WHO/PSI1 that has been translated into Chinese and validated for content validity and test-retest reliability (0.85) and then back translated into English for verification of the accuracy of translation. The degree of anxiety was divided into the following five categories: not at all, a little bit, moderate, quite a bit and extreme [24]. Before the formal follow-up period started they all were asked to finish it. After that in the follow-up period, if they were attacked by patients or colleagues in the working environment and felt hurt, they should use the Internet to complete three report forms: the Event Form, the Victim Form, and the Perpetrator Form. The Event Form contains 12 questions asking about the type of violence, its severity, date, time, the situation, *etc.* The Victim Form contains 17 questions asking about the psychological impact, how to call for help, how to prevent such an event, *etc.* The Perpetrator Form includes 20 questions asking about who conducted the violence, (a patient, a co-worker, or a supervisor). All these forms were designed as a simple-click pattern, and it only took about three minutes to finish a report, if they did not complete all the questions before they sent out the data, the screen would return to the questions left blank and prompt for completion. They were regularly reminded by a study assistant in the ward to ensure that they routinely and consistently reported any assaults. In addition, to console the victims, the assistant gave them a gift worth about 2–3 US dollars after they reported the event. Automatically, all the data we collected from website could be transferred to a database, which we could use for statistical analysis.

### 2.3. Statistic Analysis

Every violent event was collected and classified into one of the following five different categories: physical violence (PV), verbal abuse (VA), bullying/mobbing (BM), sexual harassment (SH), and racial harassment (RH), and a clear definition for each type of violence was given to the reporter before they report. The incidence rates for different types of violence were calculated separately. Because violence events were usually rare and suitable for Poisson distribution, we used the following model as the analysis framework, ln(E(Y)) = *β*_0_ + *β*_1_ × Age + *β*_2_ × Sex + *β*_3_ × Education level + *β*_4_ × Marital status + *β*_5_ × Religious belief + *β*_6_ × Duration of employment + *β*_7_ × Place of working + *β*_8_ × Degree of anxiety, where E(Y) denotes the expected number of violence reported and rate ratio (RR) equals to e*^β^* (RR_i_ = e*^β^*^i^). And we estimated the crude rate ratio (CRR) for each variable in PV, VA, BM, SH and adjusted rate ratio (ARR), 95% confidence interval (95%CI) for PV and VA to identify the significant difference between the subgroups within each variable, e.g., <30 y/o *vs*. 30–44 y/o.

## 3. Results

During the follow-up duration of one year, from 1/9/2005 to 31/8/2006, a total of 167 cohort members completed this study, three of them resigned and others retired, and dropped out from this study as early as 2 or 3 months; the drop-out rate was 0.04, and there were no violence reported by them. A total of 971 events were reported.

Table 1 manifests the demographics of the 167 cohort member. There was no missing data in the table, and about one quarter of members was aged less than 30, and about one third were male and religious. Most were college educated, married, and working in acute ward. The mean of duration of employment was 7.6 (SD 7.1, minimum 0, maximum 31) years. More than half of them reported having “a little bit” anxiety level about workplace violence.

Table 2 shows the frequency distribution of the 971 violent events reported, and the incidence rate of PV, VA, BM, and SH, 1.7, 3.7, 0.2, and 0.3 per staff-year, respectively. No racial harassment was reported in this study. Because only seven cases dropped out as early 2–3 months as the study began and no violence were reported by them, we simply neglected their contributions to calculate the incidence. For example, to get the incidence of total violence, the number of total violence reported is divided by the number of staff completed the study and the follow-up time, 1 year, that is, incidence of total violence = 971/(167 × 1) = 5.8 per staff-year (Table 2). If we considered the drop-out cases, three of them dropped out on 31/10/2005, and four dropped out on 30/11/2005, then the incidence of total violence would be gained by 971/[(167 × 1) + 3 × (2/12) + 4 × (3/12)] = 5.76, only a little less than 5.8.

Table 3 shows that age younger than 30 and elder than 44, female, college educated, unmarried, shorter duration of employment, acute and chronic ward working, and higher level of anxiety seems to be the risk factors of PV, while age younger than 30 and elder than 44, female, unmarried, religious, shorter duration of employment, chronic ward and higher level on anxiety are the risk factors of VA. Due to the small events reported in this study we could not get the adjusted rate ratio (ARR) and only part of the risk factors of BM and SH revealed significant estimate. The young age, female, college educated, unmarried, shorter duration of employment and religious are the risk factors of BM, and female, college educated, shorter duration of employment, acute ward and moderate worry, the risk factors of SH.

## 4. Discussion

In this study, using an Internet website for cohort members to report workplace violence is a new and good beginning. It is very convenient in Taiwan to use computers so we utilized the computer for reporting the violence. Another benefit of a computer-based reporting system was that the data we collected from computer could be used directly for data analysis, and the coding procedure was unnecessary. Besides, the missing data could be diminished through the computer designation, as we can see that there is no missing data shown in Table 1. It was certainly more effective and accurate than the paper-based data collection [22]. This paper focused on the risk factors of staffs, and found that higher anxiety level, especially the group of a little worry and moderate worry, was the risk factor for most of the violence. This finding corroborated the previous questionnaire survey which showed the higher anxiety level was related to higher prevalence of workplace violence [24]. The explanation of this result might be the anxiety of staffs could provoke patient’s emotion and in turn to a violence behavior. The result that the group with very, or extremely worry did not show as a statistically significant risk factor might be due to the small sample size.

Other findings of this study were that the nursing staffs with the age younger than 30 and older than 44, female, shorter duration of employment and anxiety level were the risk groups. This finding that young age and shorter employment duration were violence risk factors was comparable with another study in Taiwan [19]. Regarding the staff aged over 44, we supposed that they had been working in this hospital since the asylum era. They are used to using authority to instruct patients and seemed more likely to encounter PV and VA [24]. The fact that female is vulnerable to violence, especially sexual harassment, was mentioned in [25,26]. Previous studies demonstrated that the demographics were unrelated to the violence [27,28]. In our study demographic data, such as education level, marital status, and religion belief were not consistent with various type of violence. But after adjustment, high school level of education, unmarried, and religious group seemed to be the risk factor of physical violence and verbal abuse. Working in acute ward was a risk factor for physical and sexual assault. So, it might be very important to control the patients’ psychotic symptom to prevent this kind of workplace violence [29].

Even though the incidence of bullying and mobbing and sexual harassment was low; these issues actually remained in this hospital. Because such kind of violence impacted the staff deeply, it is mandatory to take care of the victims [26,30–33]. Regarding that RH was not reported in this study, as we found in another study, the prevalence of RH was only 4% [24], we can only speculate that the staff in this hospital suffered this problem less.

### Limitations

Because the study participants were self-referred and encouraged by a gift worth about 2–3 US dollars for completing the online report, the number of reported incidents might be too high. However, we found that most of the psychiatric staff did not think that the violence in psychiatry wards was severe or important enough to report [29]. Thus, this study’s strategy should be a reasonable approach to determine the level of violence on a psychiatric ward.

Though the website was convenient to use and we let the participants to practice the report procedure before the study, it still presented some disadvantages. As mentioned before, some members were not comfortable using the website, and this reduced their motivation to report violence, and also would likely misrepresent staff with an age bias (older nurses being less likely to use the computer-based system). In addition, the findings of this study were limited to one hospital. It is not clear if generalizations can be made from this single study.

The study focuses on characteristics of victims of assault, but it is likely that patient, administrative, and environmental characteristics have much more impact on preventive approaches. Focusing on the victim as somewhat of a “blame-the-victim” ring, and it is pretty easy to recognize that all psychiatric workers are at pretty high risk of many types of workplace violence.

However, in spite of the above limitations, the study still provided empirical evidence that a high anxiety level may cause a worker to be vulnerable to violence. Reduction of the anxiety level may be an effective approach to reduce violence.

## 5. Conclusions

In future, pre-placement education should focus on those staff with young age, female, shorter duration of employment, working in acute ward and higher anxiety level to reduce workplace violence. The future study should focus on why these staffs experience more conflict. Does their personality or the way they are perceived or educated attribute to the reason?

## Figures and Tables

**Table 1.** Frequency distribution (n, %) of demographics of cohort members n = 167.

***Demographics***	***n***	***%***
*Age*		
*<30 y/o*	*42*	*25*
*30–44 y/o*	*78*	*47*
*>44 y/o*	*47*	*28*
*Sex*		
*Male*	*62*	*37*
*Female*	*105*	*63*
*Education level*		
*High school*	*73*	*44*
*College*	*94*	*56*
*Marital status*		
*Unmarried*	*78*	*47*
*Married*	*89*	*53*
*Religious belief*		
*Religious*	*53*	*32*
*Unreligious*	*114*	*68*
*Workplace*		
*Acute ward*	*90*	*54*
*Chronic ward*	*57*	*34*
*Rehabilitation ward*	*20*	*12*
*Degree of worry about violence*		
*Not at all*	*23*	*14*
*A little worried*	*94*	*56*
*Moderate worried*	*32*	*19*
*Very and extremely worried*	*18*	*11*

**Table 2.** Incidences of types of violence, from 1/9/2005 to 31/8/2006.

*Types of violence*	*n*	*%*	*Incidence (per staff year)*
*Physical violence*	*284*	*29.6*	*1.7*
*Verbal abuse*	*611*	*62.9*	*3.7*
*Bullying and mobbing*	*33*	*3.1*	*0.2*
*Sexual harassment*	*43*	*4.4*	*0.3*
*Racial harassment*	*0*	*0*	*0*
*Total*	*971*	*100*	*5.8*

**Table 3.** Crude rate ratio (CRR) for each variable in physical violence (PV), verbal abuse (VA), bully/mobbing (BM), sexual harassment (SH), and adjusted rate ratio (ARR), 95% confidence interval (95%CI) in PV and VA by Poisson regression analysis.

*Variables*	*PV*				*VA*				*BM*	*SH*

	*CRR*	*ARR*	*95%CI*		*CRR*	*ARR*	*95%CI*		*CRR*	*CRR*
*<30 y/o vs. 30–44 y/o*	*7.03*	*1.70*	*1.17*	*2.48*	*4.53*	*1.39*	*1.07*	*1.79*	*28.5*	
*>44 y/o vs. < 30–44 y/o*	*2.18*	*12.68*	*7.61*	*21.12*	*4.44*	*26.58*	*18.73*	*37.71*	*0.81*	
*Female vs. male*	*8.58*	*4.48*	*2.53*	*7.92*	*21.98*	*15.64*	*9.21*	*26.84*	*18.92*	*24.78*
*College vs. high school*	*1.97*	*6.11*	*9.30*	*4.01*	*0.85*	*4.71*	*5.93*	*3.74*	*24.78*	*24.78*
*Unmarried vs. married*	*8.08*	*6.05*	*4.18*	*10.38*	*6.89*	*7.92*	*6.05*	*10.38*	*35.52*	
*Religious vs. unreligious*	*1.19*	*1.55*	*1.15*	*2.12*	*3.22*	*2.61*	*2.01*	*3.39*	*3.46*	*1.11*
Short* vs. long duration of employment	*1.20*	*1.23*	*1.32*	*1.18*	*1.11*	*1.19*	*1.23*	*1.16*	*1.59*	*1.37*
*Acute vs. rehabilitation ward*	*8.67*	*7.17*	*4.35*	*11.70*	*1.08*	*1.60*	*1.09*	*2.34*		*52.98*
*Chronic vs. rehabilitation ward*	*3.74*	*5.42*	*3.35*	*8.76*	*1.80*	*6.17*	*4.44*	*8.67*		*2.72*
*A little worry vs. not at all*	*14.44*	*9.87*	*2.69*	*36.23*	*19.49*	*5.53*	*2.25*	*13.46*		*7.10*
*Moderate worry vs. not at all*	*19.11*	*16.44*	*4.39*	*62.18*	*13.20*	*4.95*	*1.99*	*12.30*		*9.68*
*Very and extreme worry vs. not at all*	*3.22*	*2.61*	*0.60*	*11.36*	*3.63*	*0.72*	*0.27*	*1.93*		

* Less than four years of duration of employment.

A blank means the sample size is too small to get the meaningful rate ratio.
